# Neuronal correlates of ketamine and walking induced gamma oscillations in the medial prefrontal cortex and mediodorsal thalamus

**DOI:** 10.1371/journal.pone.0186732

**Published:** 2017-11-02

**Authors:** Katrina E. Furth, Alex J. McCoy, Caroline Dodge, Judith R. Walters, Andres Buonanno, Claire Delaville

**Affiliations:** 1 Neurophysiological Pharmacology Section, National Institute of Neurological Disorders and Stroke, National Institutes of Health, Bethesda, Maryland, United States of America; 2 Graduate Program for Neuroscience, Boston University, Boston, Massachusetts, United States of America; 3 Section on Molecular Neurobiology, Eunice Kennedy Shriver National Institute of Child Health and Human Development, National Institutes of Health, Bethesda, Maryland, United States of America; Technion Israel Institute of Technology, ISRAEL

## Abstract

Alterations in the function of the medial prefrontal cortex (mPFC) and its major thalamic source of innervation, the mediodorsal (MD) thalamus, have been hypothesized to contribute to the symptoms of schizophrenia. The NMDAR antagonist ketamine, used to model schizophrenia, elicits a brain state resembling early stage schizophrenia characterized by cognitive deficits and increases in cortical low gamma (40–70 Hz) power. Here we sought to determine how ketamine differentially affects spiking and gamma local field potential (LFP) activity in the rat mPFC and MD thalamus. Additionally, we investigated the ability of drugs targeting the dopamine D4 receptor (D4R) to modify the effects of ketamine on gamma activity as a measure of potential cognitive therapeutic efficacy. Rats were trained to walk on a treadmill to reduce confounds related to hyperactivity after ketamine administration (10 mg/kg s.c.) while recordings were obtained from electrodes chronically implanted in the mPFC and MD thalamus. Ketamine increased gamma LFP power in mPFC and MD thalamus in a similar frequency range, yet did not increase thalamocortical synchronization. Ketamine also increased firing rates and spike synchronization to gamma oscillations in the mPFC but decreased both measures in MD thalamus. Conversely, walking alone increased both firing rates and spike-gamma LFP correlations in both mPFC and MD thalamus. The D4R antagonist alone (L-745,870) had no effect on gamma LFP power during treadmill walking, although it reversed increases induced by the D4R agonist (A-412997) in both mPFC and MD thalamus. Neither drug altered ketamine-induced changes in gamma power or firing rates in the mPFC. However, in MD thalamus, the D4R agonist increased ketamine-induced gamma power and prevented ketamine’s inhibitory effect on firing rates. Results provide new evidence that ketamine differentially modulates spiking and gamma power in MD thalamus and mPFC, supporting a potential role for both areas in contributing to ketamine-induced schizophrenia-like symptoms.

## Introduction

Patients with early stage schizophrenia display task-related impairments in cortical gamma power (40–70 Hz) modulation that are correlated with cognitive deficits [1–6]. Alterations in the relationship between the medial prefrontal cortex (mPFC) and its major thalamic source of innervation, the mediodorsal (MD) thalamus [7] may also contribute to psychotomimetic and cognitive symptoms of schizophrenia [8–10]. Evidence from post-mortem histology and fMRI studies suggests that the MD thalamus of patients with schizophrenia have fewer neurons and fewer functional connections with the mPFC than are observed in non-schizophrenia controls, although recent evidence has been mixed [for reviews, see 11–13]. Furthermore, lesions in the MD thalamus profoundly impair cognition and can lead to schizophrenia-like symptoms [14]. One strategy for studying the neurophysiological correlates of schizophrenia has involved the use of the NMDA receptor antagonist, ketamine, in rodents [15,16]. Importantly, ketamine elicits a brain state in healthy humans resembling early stage schizophrenia, characterized by cognitive impairments, increased cortical gamma oscillation power, decreased task-related gamma power, and alterations in thalamocortical relationships [17–23]. Ketamine has also been shown to affect oscillatory activity in the prefrontal cortex in rodents in multiple frequency ranges, including the gamma range [24–26]. These observations have led to the use of ketamine as a tool for examining the therapeutic potential of drugs for the treatment of schizophrenia [27,28].

Although alterations in thalamocortical relationships have been suggested to play a role in the pathophysiology of schizophrenia [29,30], changes in thalamic activity in rodent models of schizophrenia have been less well characterized than changes in prefrontal cortex. In the ventrolateral (VL) thalamus of freely moving rats, ketamine increases gamma power [31]. Similarly, in the ventral posteromedial (VPM) thalamus, ketamine increases spontaneously occurring and reduces sensory-evoked gamma oscillations [32,33]. Furthermore, local NMDA receptor antagonism in the MD thalamus is capable of reproducing the systemic effects of NMDA receptor antagonism on delta frequency oscillations in the mPFC of anesthetized rats [34]. Preclinical studies have examined the effects of NMDAR antagonists on firing rate in the mPFC [35–37] and MD thalamus [38,39]; however, the relationship between spike timing and the changes in gamma power after NMDAR antagonist administration remains uncertain [37,40]. Further insight into the neuronal mechanisms by which ketamine increases gamma power within mPFC local circuits in conjunction with parallel changes within the MD thalamus could be useful for understanding ketamine’s clinical relevancy [37,41].

The dopamine D4 receptor (D4R) has emerged as a potential pro-cognitive target in schizophrenia due to its expression in the mPFC [42], and the ability of D4R agonists to increase gamma power and cognitive performance [43–45]. Similarly, a D4R antagonist can reverse cognitive deficits induced by stress or chronic NMDA receptor antagonism [46,47]. However, the effects of a D4R agonist and antagonist on gamma power and single neuron activity in the ketamine model of early schizophrenia remain unknown.

This study sought to determine how an acute subanesthetic dose of ketamine differentially affects spiking and gamma oscillation activity in the mPFC and MD thalamus in rats. Recordings were performed while rats walked on a rotating circular treadmill because ketamine has been shown to induce hyperlocomotion in rodents separate from its effects on gamma activity [31]. The walking task regularized motor activity before and after ketamine administration and a stationary paddle across the track ensured that rats maintained an alert, attentive state. As decreases in MD thalamus activity in combination with changes in mPFC activity are correlated with the emergence of schizophrenia symptoms [14], we predicted that ketamine-treated rats would demonstrate reductions in spiking and changes in LFP activity in the MD thalamus in conjunction with changes in mPFC activity. Additionally, we administered an agonist and antagonist of the dopamine D4R to examine their acute effects on the LFP and single neuron activity of the MD thalamus and mPFC in ketamine-treated rats to look for changes in gamma activity consistent with therapeutic potential.

## Materials and methods

All procedures were conducted in accordance with the National Institutes of Health Guide for Care and Use of Laboratory Animals and approved by National Institute of Neurological Disorders and Stroke animal care and use committee. We attempted to minimize the number of animals used and their discomfort.

After completion of surgeries, 0.15% of ketoprofen in 0.9% NaCl solution was given subcutaneously to minimize the rats' discomfort. During the first week of postoperative recovery, the rat’s diet was supplemented with fruit and bacon treats, and animals were observed three times a day for any signs of discomfort. Further doses of 0.15% of ketoprofen in 0.9% NaCl solution were given only if the animal showed signs of discomfort.

### Rats and behavioral paradigm

**Fig 1:**
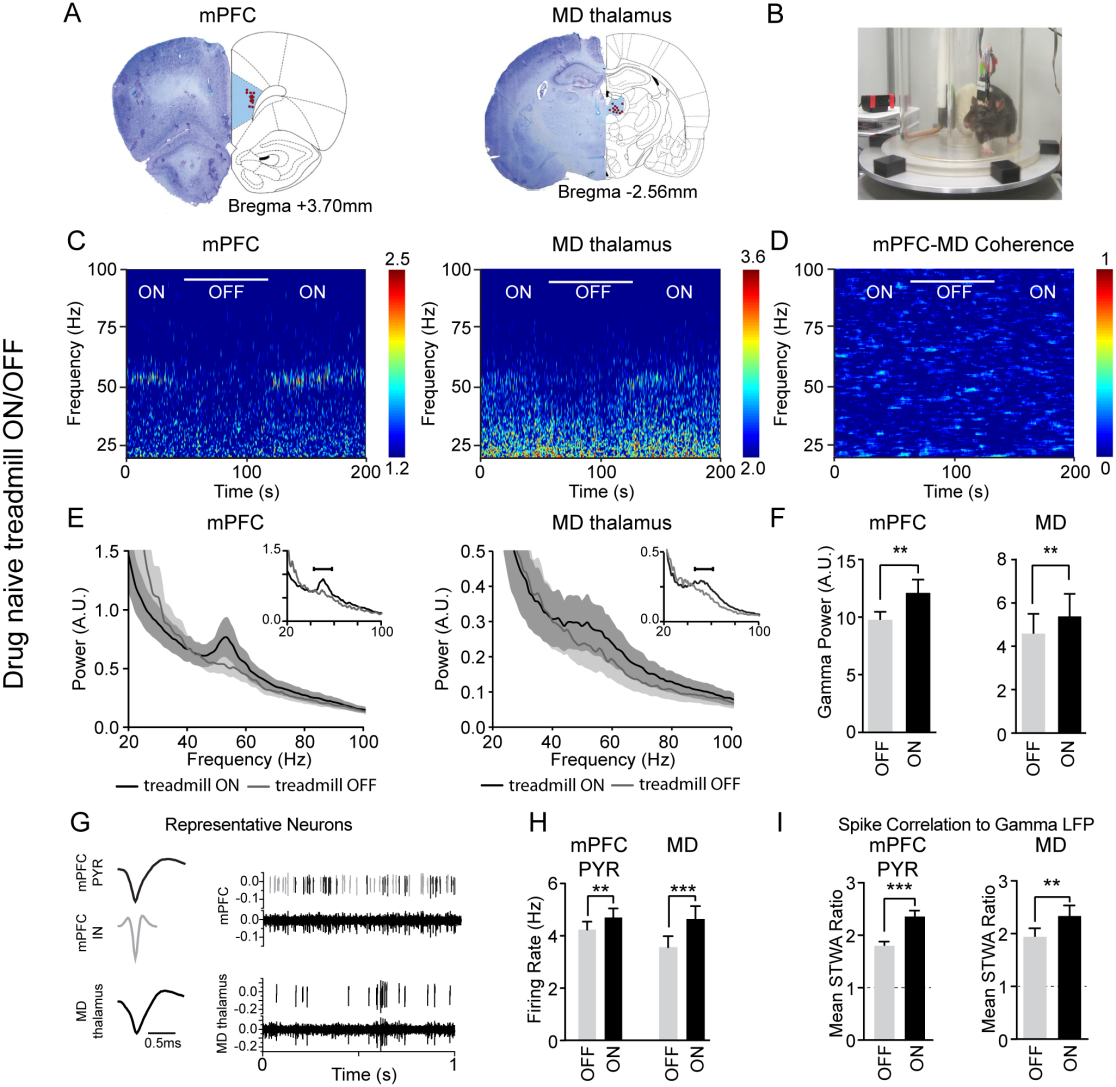
Walking-induced increases in gamma power, firing rates and spike-gamma LFP correlations. **A,** Histological reconstruction showing locations (red dots) of the recording electrodes in the prelimbic mPFC and MD thalamus. **B,** Photograph shows the rotating circular treadmill. The paddle in the back ensured that the rat continued walking. **C,** Representative wavelet-based scalograms show time-frequency plots of normalized LFP spectral power during epochs with the treadmill turned ON or OFF (white bar). Warmer colors indicate higher power. **D,** Representative FFT-based spectrogram shows time-frequency coherence between the mPFC and MD thalamus. **E,** Average LFP power spectra ± SEM for epochs with treadmill ON (black) or OFF (grey) on day 1 of recording (n = 12 rats). Insets show representative traces for each structure. **F,** Bar graphs shows mean total gamma LFP power with the treadmill ON and OFF from day 1 of recording (n = 12 rats). The gamma frequency range was determined using the 15 Hz surrounding the significant gamma peak (see Methods) between 40 and 70 Hz in each treadmill walking epoch. **G,** Representative pyramidal neuron (PYR, black) and interneuron (IN, grey) waveforms, raw signals and spike trains from the mPFC and MD thalamus during treadmill walking. **H**, Firing rates in the mPFC of PYR neurons (n = 118 neurons from 12 rats), and from MD thalamus neurons (n = 62 neurons from 12 rats). **I,** Mean ratios between peak-to-trough amplitudes of the original spike-triggered waveform average (STWA) and the mean of 20 shuffled STWAs for LFPs filtered from 40–70 Hz recorded from a neighboring wire (see Methods). A ratio of 1 (dashed horizontal line) indicates no difference between shuffled and unshuffled values. Values are reported as mean ± SEM. *: *p* < 0.05, **: *p* < 0.01, ***: *p* < 0.001, bootstrap tests.

Male Long Evans rats (Charles River, Frederick, MD, USA), weighing 280–300 g, were housed with ad libitum access to chow and water in environmentally controlled conditions with a 12:12 h light:dark cycle (lights off at 9:00 AM). Rats were handled daily the week before surgery and trained to walk on a circular treadmill (Fig 1B) [48].

### Surgical procedures

Rats were anesthetized with 75 mg/kg ketamine and 0.5 mg/kg medetomidine (intraperitoneal, i.p.) and placed in a stereotaxic frame (David Kopf Instruments, Tujunga, CA, USA), and head-fixed with atraumatic ear bars. Holes were drilled in the skull above the target coordinates for the mPFC (AP: +3.6 mm from the bregma, ML: +0.6 mm from the sagittal suture and DV: 3.6 mm from the skull surface; Fig 1A), and MD thalamus (AP: -2.5 mm from the bregma suture, ML: +0.5 mm from the sagittal suture and DV: 5.8 mm from the skull surface; Fig 1A). Electrode bundles (NB Labs, Denison, TX) consisted of 8 stainless steel teflon-insulated microwires plus an additional 9^th^ wire with no insulation on the distal ~1 mm of the recording tip serving as a local reference [49–51]. The electrode bundles were implanted in the target regions and secured to the skull with screws and dental cement. Ground wires from each set of electrodes were wrapped around a screw located above the cerebellum. After completion of surgeries, 0.15% of ketoprofen in 0.9% NaCl solution and atipamezole (0.3–0.5 mg/kg) were administered subcutaneously to reverse the effect of medetomidine.

### Behavioral analysis

Behavioral data synchronized with electrophysiological recordings was recorded from a web camera (Logitech) mounted next to the circular treadmill. A blinded experimenter assessed the rodent’s behavior using 50-second video epochs with the treadmill off and on. Behavior with the treadmill on was scored from one epoch taken before the ketamine injection (baseline) and another 15 minutes after the ketamine injection. Treadmill-off epochs were selected from baseline and 13 minutes after the ketamine injection.

With the treadmill off, the rat stayed inside the circular treadmill track (Fig 1B). In this condition, untreated rats entered states of rest, but ketamine-treated rats were either very active, or ataxic. To evaluate motor activity under these conditions, we measured the number of seconds that the rat spent moving its trunk or limbs but not its head. Ataxia was scored according to the following scale [adapted from 52]: (0) inactive or coordinated movements, (1) jerky movements or loss of balance while rearing, (2) partial impairment of antigravity reflexes, (3) the inability to move beyond a small area and support body weight and (4) inability to move except for twitching movements. To measure stereotypic behaviors, we quantified the number of head-bobs, turns, and the number of seconds spent backpedaling [adapted from 52]. Head-bobs included side-to-side or vertical head movements, and turns involved a 180 degree turn so that the rat’s direction in the treadmill had changed.

During treadmill-on epochs, a blinded experimenter recorded the number of turns, the number of times that the rat hit the paddle, and the length of time the rat spent being pushed by the paddle. Paddle hits counted for any of the rat’s body parts other than the tip of his tail.

### Electrophysiological recordings

Voltage signals from the eight electrode wires within each electrode bundle were referenced to a scraped 9^th^ wire in the same bundle. Broadband electrophysiological signals were amplified and discriminated into extracellular spike trains and LFPs with high- and low-pass filters, respectively, using Plexon pre-amplifiers (Dallas, TX). High-pass filtered spike channels and low-pass filtered LFP channels were sampled with CED Micro1401 data acquisition interface (Cambridge Electronic Design, Cambridge, UK). Sampling rates were 40 kHz for spike trains and 2 kHz for LFPs. High-pass filtered waveform channels were amplified (10,000X) and bandpass filtered (0.3–8 kHz). Low-pass filtered LFP channels were amplified (1,000X) and bandpass filtered (0.7–150 Hz). Discriminated spike signals and LFPs recordings were digitized, stored and analyzed offline using Spike2 software (Cambridge Electronic Design, Cambridge, UK).

Baseline recordings consisted of at least two five-minute epochs of counterclockwise walking with the treadmill rotating slowly and two 40 second epochs of inattentive rest without artifacts. After drug injections, the rat was placed on the treadmill, then the treadmill was turned on for 4 minutes, and off for one minute. This pattern was repeated five times for the first 25 minutes. Then the treadmill was turned off for the final 4 minutes before the next injection. Direct observation and videotaped motor behavior were used to identify artifact-free 100 second intervals within the treadmill-on epochs and 40 to 100 second intervals from treadmill-off epochs.

### Experimental conditions and drugs

Starting one week after the surgery, recording sessions were conducted once a week with at least six days between recordings (S1 Table). Table 1 shows the time course of drug injections. Treadmill on and off recordings at post-surgical day 7 were obtained from 15 rats.

**Table 1:** Time course of treatments.

Experiment	Injection 1 Time = 0 min	Injection 2 Time = 30 min	Injection 3 Time = 60 min	Injection 4 Time = 90 min
D4R dose response curve (n = 6)	0.3 mg/kg A-412997	1.2 mg/kg A-412997	1.5 mg/kg A-412997	5 mg/kg L-745,870
Vehicle dose response curve (n = 6)	1 mg/mL saline	1 mg/mL saline	1 mg/mL saline	5 mg/kg L-745,870
Double doses of ketamine (n = 8)	5 mg/kg ketamine	1 mg/mL saline*	5 mg/kg ketamine	
Vehicle pretreatment + Ketamine (n = 6)	1 mg/mL saline	10 mg/kg ketamine		
L-745,870 pretreatment + Ketamine (n = 6)	5 mg/kg L-745,870	10 mg/kg ketamine		
A-412997 pretreatment + Ketamine (n = 6)	3 mg/kg A-412997	10 mg/kg ketamine		
Final ketamine	90 or 100 mg/kg ketamine	0.15 mg medetomidine#		

Each recording consisted of a 30 minute drug-free baseline followed by a 125 minute post injection period. The time course of the injections is indicated in the table. Recordings were collected once a week with at least six days between recordings

*injected at 45 minutes. See S1 Table.

^#^ injected between 20 and 30 minutes.

Rats that participated in the D4R dose response curve experiments were randomly assigned to receive the vehicle or D4R agonist first (n = 6). The D4R agonist A-412997 (Tocris, MN) and antagonist L-745,870 (Tocris, MN) were dissolved in saline and injected at 1 mL/kg subcutaneously. Doses of the agonist were chosen based on Kocsis and colleagues [53] so that the final total dose was 3 mg/kg of A-412997. Injections were given every 30 minutes in ascending order from 0.3 mg/kg through 1.5 mg/kg A-412997, as described in Table 1.

In the final experiment, we employed a drug testing paradigm like the one used by Jones and colleagues [54] in which a pretreatment was administered 30 minutes before an injection of ketamine (n = 6, Table 1). In short, we recorded 30 minutes of baseline, followed by an injection of one of three pretreatments—saline, 3 mg/kg A-412997, the D4R agonist, or 5 mg/kg L-745,870, the D4R antagonist. Each drug was administered to every rat according to a random design. Thirty minutes after the pretreatment, a single injection of 10 mg/kg ketamine was given subcutaneously, and recordings continued for the next 100 minutes. The final anesthetized recording was combined with the last experimental recording in about half of the rats and started 100 minutes after the ketamine injection. If the rat had already received 10 mg/kg ketamine, then the final dose was 90 mg/kg. Otherwise 100 mg/kg of ketamine was administered subcutaneously as the final ataxia-inducing dose.

### Spectral analysis of local field potential recordings

LFP power was measured by fast Fourier transform (FFT) with a frequency resolution of ~1 Hz and normalized by dividing the power at each frequency by the sum of power between 250 and 300 Hz in order to compensate for any instrumental fluctuations over time [55]. For each structure, total power was calculated two different ways: (1) using a pre-specified range for low gamma (40–70 Hz) or (2) for each recording, a significant gamma peak was determined, and power was summed 7 Hz below and above the peak to find the total gamma power. A peak was considered significant if its relative maximum was greater than the surrounding 14 frequency bins in the low gamma range, the first derivative of the spectrum was positive to the left of the peak and negative to the right of the peak, and the second derivative at the peak was negative, indicating a downward concavity. LFP power from two wires per electrode bundle during two epochs was averaged for each behavioral condition. Spectral coherence was calculated for the same set of wires between the mPFC and MD thalamus using a Spike2 script and FFT-based spectral coherence. Coherence was calculated, and a lower limit line of coherence significance was constructed using the following formula, where L is the length of the epoch [56]:

$$\text{Coherence}\;\text{significance}\;\text{line}=1-{\left(\text{p-values}\right)}^{1/(\text{L}-1)}$$

Using 100 second epochs, any coherence over 0.015 was considered significant [56]. Data are reported as mean ± SEM.

Time-frequency wavelet spectra were constructed using continuous wavelet transforms. The Morlet wavelet was applied to the LFPs using 128 frequency scales and a time resolution of approximately 750 ms (Time-Frequency Toolbox (http://tftb.nongnu.org). Time-frequency mPFC-MD thalamus coherence was calculated using FFT-based coherence over a 10 second sliding window (Chronux: http://chronux.org).

The multitaper coherence analysis introduced a bias determined by the following formula:

$$B=\frac{1}{\sqrt{\nu }}$$

*B* is the bias introduced by the analysis and *ν* represents the degrees of freedom, equivalent in this case to the number of tapers used. This multitaper calculation utilized 19 tapers, resulting in a bias of about 0.23. To compensate for this, the smallest coherence values in the plot were adjusted to 0.23 and the largest to 1.23, rather than 0 and 1, respectively [57].

The results of coherence should be interpreted carefully, and preferably corroborated by alternative methods. One such method is the computation of a phase difference histogram, which does not assume stationarity and allows for cross-frequency interactions [58]. To construct these phase difference histograms, the Hilbert transform of gamma-filtered LFPs (40–70 Hz) was calculated to find the instantaneous phase of each waveform. The instantaneous phases for one LFP were then subtracted from the instantaneous phases of the other LFP and the resulting differences were plotted as a histogram. These polar histograms were reviewed in conjunction with the coherence analysis.

### Cell sorting and spike-triggered waveform analysis

Spike trains were generated from broadband signals by first extracting spikes and then by sorting spikes into putative single-unit clusters via principal components analysis (PCA) in Spike2. The channels containing spiking information were high-pass filtered a second time using a cutoff of 300 Hz and a transition gap of 100 Hz to guarantee that channels were free of low frequency content. The RMS amplitudes of the high-pass filtered channels were then measured using Spike2; these RMS amplitudes were multiplied by a factor of 4 to yield the minimum threshold for extraction of spike waveforms from the continuous data. Spike waveforms were extracted from the continuous data using the 4xRMS threshold, plotted using PCA, and clustered using the Normal Mixtures clustering algorithm.

To assess effective sorting for single cells, interspike interval histograms were generated and inspected to ensure that sorted cell clusters did not exhibit multiunit behavior by verifying the absence of multiple spikes within the assumed refractory period (1.2 ms). Putative cortical pyramidal neurons and interneurons were separated using trough-to-peak intervals [49,59,60]. Units with trough-to-peak intervals greater than 0.5 ms were classified as putative pyramidal neurons (112/147 neurons), and those with intervals less than 0.4 ms were classified as interneurons (31/147 neurons). However, only two of these 31 interneurons could be detected by the time ketamine was first administered, two to three weeks later, so the further analysis focused solely on pyramidal neurons. Four neurons did not fit into either category and were left unclassified. In this paper, the terms pyramidal neuron and interneuron refer to putative pyramidal and putative interneurons, respectively.

To assess the temporal relationship between the spiking activity of individual neurons from one wire (“same-wire”) and LFPs recorded from a neighboring wire within the same bundle, spike-triggered waveform averages (STWAs) were calculated for epochs of 100 to 200 seconds. The “neighboring wire” was identified by obtaining LFP coherences between the wire on which the spike train was recorded (“same-wire”) and all other wires within the electrode bundle. The wire with the maximum LFP coherence to the same-wire was selected as the “neighboring wire” and used for determining STWA data. The average coherence between all same-wire LFPs and neighboring wire LFPs was 0.73 ± 0.01 (n = 112). LFPs from the neighboring wire were band-pass filtered between 40 and 70 Hz. Peak-to-trough amplitudes of each unit’s STWA at or around the spike (zero time) were obtained as a measure of correlation between the spikes of each neuron in the spike train and the phase of the dominant gamma oscillation. Twenty additional “shuffled” STWAs were created for each of the single units by shuffling the interspike intervals in the same epochs twenty times to provide 20 randomized spike trains. The STWAs generated from each of the 20 randomized spike trains provided a set of normally distributed peak-to-trough values to compare to the original STWA from unshuffled data. The extent of correlation between spikes and gamma-filtered LFPs after ketamine or D4R drug treatments during epochs of walking was compared to baseline walk epochs using the mean ratios of unshuffled to shuffled peak-to-trough amplitudes (mean STWA ratio).

### Statistics

We used non-parametric bootstrap with replacement to calculate confidence intervals for analysis of LFP power, spectral coherence, firing rate, and mean STWA ratios. Effects were considered significant when the confidence interval for the difference in means did not contain 0. For paired observations, the difference between baseline and the test condition was first calculated. These values were then bootstrapped 10,000 times with replacement, and compared to 0 to determine significance. Whenever multiple comparisons were performed using the same data, the significance level of α = 0.05 was corrected using a Bonferroni correction. A permutation test was used to show that mean STWA ratios were significantly different from a randomly ordered spike train (with a theoretical value of 1). Examination of gamma power across time was assessed using two-way repeated measures analysis of variance (ANOVA) with the level of significance: α = 0.05. Outlier time points for power were removed using the ROUT (robust nonlinear regression combined with outlier removal) test with Q set to 1% [61] and the average of the other rats was used as a replacement value [51]. Outlier firing rates and STWA ratios were removed using the ROUT test with Q set to 0.1% [61].

### Histology

After recordings were completed, rats were deeply anesthetized with ketamine (100 mg/kg s.c.) and 0.15 mg medetomidine and recording sites were marked by passing a positive current via 3 microwires and the scraped reference microwire. Rats were perfused intracardially with 200 mL cold saline followed by 200 mL 4% paraformaldehyde in phosphate buffered saline (PBS). Brains were post-fixed in paraformaldehyde solution overnight and then immersed in 10% sucrose in PBS (0.1 M, pH 7.4). Coronal sections of 35 μm were collected on slides. Sections for electrode placement verification were mounted on glass slides and stained with cresyl violet and 5% potassium ferricyanide-9% HCl to reveal the iron deposited at the electrode tips. Rats were only included in the analysis if the electrodes were properly placed in the prelimbic mPFC and the core or the shell of the MD thalamus.

## Results

To investigate the effects of ketamine on the relationships between behavior, gamma power and neuronal activity in the mPFC and the MD thalamus, we recorded simultaneously from these two structures before and after injection of ketamine (10 mg/kg s.c.).

### Effects of treadmill walking on gamma power and neuronal activity

To better understand the neurophysiological effects of ketamine, we first examined the effects of treadmill walking on LFP and neuronal activity in mPFC and MD thalamus in drug naïve rats; then we examined the effects of ketamine administration before and during treadmill walking. Previous work in our lab has shown that low gamma power in the mPFC and subthalamic nucleus increases during motor activity compared to periods of quiet rest [50]. Current results confirmed that treadmill walking alone increases mPFC low gamma power. During treadmill-off epochs, seven days after electrode implantation, there was no significant spectral peak in the low gamma range (40–70 Hz). In contrast, during treadmill-on epochs, when the rats were walking steadily, the power spectra of the mPFC showed an increase in a relatively narrow band with a mean peak of 51.1 ± 0.8 Hz (Fig 1C & 1E). The LFP power in a 15 Hz range surrounding this significant gamma peak (see Methods) increased by 23.8% relative to treadmill-off (*p* < 0.01, paired bootstrap test, n = 12 rats; Fig 1F). Interestingly, in the MD thalamus, the low gamma power increased by 17.5% (*p <* 0.01, paired bootstrap test, n = 12 rats) during treadmill walking in a broader band than observed in the mPFC, with a significantly lower frequency peak at 48.6 ± 0.5 Hz (*p* < 0.05, bootstrap test; Fig 1C, 1E & 1F). Coherence between the mPFC and MD thalamus (peak between 40 and 70 Hz ± 7 Hz) was not significant (n = 9 rats; Fig 1D).

To examine the differences between mPFC pyramidal neurons and interneurons with respect to firing rates and spike-gamma LFP correlations, we analyzed waveform shape and firing rates for all well-isolated neurons [62] (see Methods, Fig 1G). Analysis was focused on putative pyramidal neurons as interneurons were only sparsely detected. Overall, the firing rates of mPFC pyramidal neurons and MD thalamus neurons increased during treadmill walking. The firing rates of pyramidal neurons in the mPFC, increased by 12% from 4.2 ± 0.3 Hz to 4.7 ± 0.3 Hz with the treadmill-on compared to treadmill-off (*p* < 0.01, paired bootstrap test, n = 116 neurons; Fig 1H). Although this average increase was modest, 40% of pyramidal neurons in the mPFC showed more than a 20% increase in firing rate, while 16% showed more than a 20% decrease with the treadmill on compared to off. Similarly, the firing rate of MD thalamus neurons also increased by 29% from 3.4 ± 0.4 Hz to 4.4 ± 0.4 Hz during treadmill walking (*p* < 0.001, paired bootstrap test, n = 62 neurons; Fig 1H).

Spike-LFP synchronization (phase-locking) in the low gamma range increased in both the mPFC and MD thalamus with the treadmill on compared to off. The extent of correlation between spikes and simultaneously recorded gamma LFPs (filtered at 40–70 Hz) was evaluated by obtaining the peak-to-trough value of the original spike triggered waveform average (STWA), and comparing it to distribution of 20 STWAs generated by randomly shuffling the interspike intervals from the same epoch (mean STWA ratio, see Methods). Specifically, mPFC pyramidal neuron spike-gamma LFP correlations increased ~31% during walking relative to epochs with the treadmill off (mean STWA ratio 2.36 ± 0.11 vs. 1.79 ± 0.08, *p* < 0.001, paired bootstrap test, n = 123 neurons; Fig 1I). Additionally, MD thalamus spike-gamma LFP correlations increased ~29% during treadmill walking (mean STWA ratio 3.08 ± 0.34 vs 2.40 ± 0.26, *p* < 0.01, paired bootstrap test, n = 70 neurons; Fig 1I). In summary, walking caused concurrent increases in gamma power and firing rates and spike-gamma LFP correlations in both the mPFC and MD thalamus.

### Effects of ketamine on gamma power and motor activity

Motor activity was scored during treadmill-off epochs as described in the methods and increased over the first 30 minutes following ketamine administration. Specifically, rats spent 2.6% of an epoch moving on average before ketamine and 91.6% of an epoch moving after ketamine (*p <* 0.05, paired bootstrap tests, n = 12 rats; S2 Table). However, ketamine increased gamma power more than would be expected given the increase in motor activity (Fig 1G) [31]. Interestingly, with the treadmill off, after ketamine administration, rats became more likely to walk backwards, turn within the track, and show stereotypic behaviors compared to baseline (all *p* < 0.05, paired bootstrap tests; S2 Table). But, with the treadmill on, ketamine administration did not significantly increase these behaviors relative to baseline (all *p* > 0.05, paired bootstrap tests; S2 Table). Furthermore, rats appeared more attentive to the walking task following ketamine administration, as they made contact with the paddle less often (*p* < 0.05, paired bootstrap test; S2 Table). Taken together, the rat’s behavior was more consistent before and after ketamine administration when the treadmill was on.

**Fig 2:**
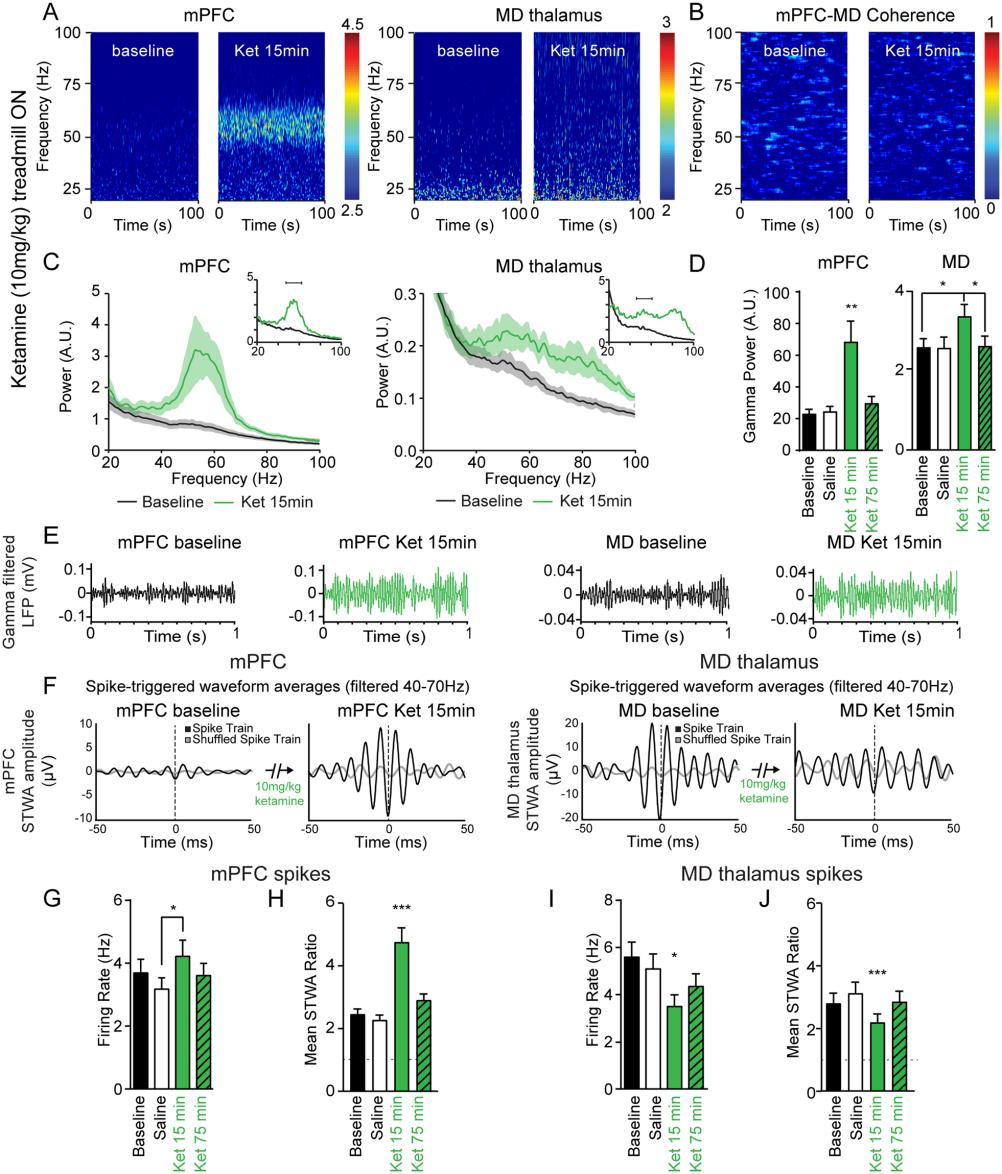
Ketamine-induced increases in gamma LFP power and spiking activity during treadmill walking. **A,** Representative wavelet-based scalograms show the time-frequency plots of LFP spectral power before ketamine (**baseline**) and 13–17 minutes after 10 mg/kg ketamine s.c. (**Ket 15 min**). **B,** Representative FFT-based spectrogram shows time-frequency coherence between the mPFC and MD thalamus before ketamine and after ketamine. **C,** Averaged LFP power spectra for epochs before ketamine (black), and after ketamine (green). Insets show representative traces for each structure. **D,** Bar graph shows mean total gamma LFP power (peak ± 7 Hz) from the mPFC (n = 10 rats) and the MD thalamus (n = 6 rats) during treadmill walking at baseline (black), 15 minutes after saline injection (white), and 15 minutes (green) or 75 minutes (green with diagonal stripes) after 10 mg/kg ketamine injection. **E,** Representative examples of gamma band-pass filtered LFP (40–70 Hz) in the mPFC and MD thalamus. **F,** Representative STWAs of a mPFC pyramidal neuron (**D**) and MD thalamus neuron (**E**) with LFPs filtered from 40–70 Hz 15 minutes before and after ketamine administration. **G, I**, Mean firing rates of pyramidal neurons in the mPFC (**G**, n = 45 neurons from 6 rats) and the MD thalamus (**I**, n = 37 neurons from 6 rats). **H, J,** Bar graphs show spike-gamma LFP correlations (filtered from 40–70 Hz) for putative mPFC pyramidal neurons (**H**) and MD thalamus neurons (**J**). Values are reported as mean ± SEM. Stars over a single bar indicate this value differs significantly from the three other conditions. *: *p* < 0.05, **: *p* < 0.01, ***: *p* < 0.001, paired bootstrap tests with Bonferroni correction.

To address the confounding effect of increases in motor activity induced by ketamine administration, we limited subsequent analysis to epochs with the treadmill on. Ketamine-induced increases in gamma power were not significantly greater in the mPFC or the MD thalamus with the treadmill on compared to off (both *p* > 0.05, paired bootstrap tests). Fifteen minutes after the ketamine injection, gamma power (peak ± 7 Hz) increased in the mPFC and MD thalamus by 254% and 31%, respectively, compared to baseline (both *p* < 0.05, paired bootstrap tests; Fig 2A, 2C & 2E). The peak frequency of the ketamine-induced gamma in the mPFC was centered at 55.8 ± 1.4 Hz (n = 6 rats). In the MD thalamus two broader peaks emerged centered around 52.4 ± 1.4 Hz and 80.8 ± 1.1 Hz (n = 6 rats; Fig 2A, 2C & 2E). The frequency of the low gamma peaks in these two structures were not significantly different (*p* > 0.05, bootstrap test).

Next, we examined the change in gamma power over time. Low gamma power (peak ± 7 Hz) was significantly higher 15 minutes after a 10 mg/kg ketamine injection in both the mPFC (+250%) and MD thalamus (+30%) compared to baseline (both *p* < 0.05, paired bootstrap tests; Fig 2D). Interestingly, gamma power was not significantly different from baseline 75 minutes after ketamine administration in the MD thalamus (*p* > 0.05, paired bootstrap test; Fig 2D). To ensure that increases in gamma power had not reached a ceiling after 10 mg/kg ketamine, we also examined the effects of 100 mg/kg ketamine. Indeed, this dose induced a robust increase in gamma power in both structures (mPFC gamma power +347% over baseline, *p* < 0.01, n = 8 rats, MD gamma power +86% over baseline, *p <* 0.01, n = 5 rats, paired bootstrap tests, data not shown); although it also induced severe ataxia by 10 min after injection.

Although the coherence showed modest increases after ketamine and reached significance in two gamma frequency ranges from 48.8–53.7 Hz and 56.6–66.4 Hz (S1 Fig), average LFP coherence between mPFC and MD thalamus in the low gamma frequency range (peak between 40–70 Hz ± 7 Hz) was not significantly increased after ketamine administration (*p* > 0.05, average coherence of 0.008 ± 0.001 at baseline vs. 0.027 ± 0.016 fifteen minutes after ketamine, paired bootstrap tests, n = 5 rats; Fig 2B). We further compared the coherence values before and after ketamine administration in each frequency bin using multiple t-tests and found no significant differences in any frequency range (False Detection Rate approach of Benjamini and Hochberg with Q = 1%; each row analyzed individually). Given that oscillations can exhibit intermittent synchronization, and that spectral coherence can fail to appear significant despite phase coordination, we also analyzed the instantaneous phase differences between the mPFC and MD thalamus to examine coherence and test for bimodal phase relationships in mutually interacting networks with conduction delays [58]. Results confirmed our previous observation that gamma frequency LFP synchronization did not increase after ketamine administration (S2 Fig).

These behavioral and electrophysiological results show that ketamine administration can induce robust increases in gamma oscillation power during treadmill walking in the MD thalamus and mPFC, two to ten times greater than those predicted by increases in motor activity. Moreover, gamma power showed a dramatic increase in a narrow band in the mPFC, and a modest increase over a broader band in the MD thalamus.

### Effects of 10 mg/kg ketamine on single neurons in the mPFC and MD thalamus

The current data show that walking and ketamine administration have similar effects on mPFC spike rate and spike-LFP synchronization, but opposite effects on these measures in MD thalamus neurons. Fifteen minutes after 10 mg/kg ketamine, the mean firing rate of mPFC pyramidal neurons was 33% higher than after saline injection (*p* < 0.05, from 3.3 ± 0.4 Hz to 4.4 ± 0.5 Hz, paired bootstrap test, n = 45 neurons; Fig 2G). In contrast, the firing rates of neurons in the MD thalamus decreased by 40% fifteen minutes after ketamine administration (*p* < 0.01, from 5.9 ± 0.7 Hz to 3.9 ± 0.5 Hz, paired bootstrap test, n = 37 neurons; Fig 2I) despite a concurrent, significant increase in low gamma power. Firing rates returned to baseline levels 75 min after ketamine injection in the mPFC (*p* > 0.05, paired bootstrap test; Fig 2G & 2I). In summary, ketamine administration has significant, yet opposite effects on the mean firing rates of mPFC pyramidal neurons and MD thalamus neurons.

Next, we examined ketamine’s effects on the synchronization between neuronal spiking and gamma frequency oscillations using spike-gamma LFP correlations. Representative STWAs from unshuffled and shuffled interspike intervals shown in Fig 2F demonstrate that spike-gamma LFP correlations increased after ketamine administration in the mPFC, but decreased in the MD thalamus. Indeed, in the mPFC, the spike-gamma LFP correlation of pyramidal neurons increased 15 minutes after ketamine, relative to baseline, saline injection, or the period 75 minutes after the ketamine injection (all *p* < 0.001, paired bootstrap tests, n = 45 neurons; Fig 2H). After ketamine administration, the extent of correlations between mPFC pyramidal neuron spikes and gamma LFPs doubled relative to baseline (+94%, mean STWA ratio = 4.77 ± 0.46 vs 2.46 ± 0.17, *p* < 0.001, paired bootstrap test; Fig 2H).

A very different picture emerged in the MD thalamus after ketamine administration. Although gamma power increased in the MD thalamus 15 minutes after 10 mg/kg ketamine, MD thalamus neuron spiking was ~22% less correlated to the MD thalamus gamma LFPs than during baseline walk (mean STWA ratio = 2.20 ± 0.27 vs 2.80 ± 0.33, *p* < 0.001, paired bootstrap test, n = 39 neurons; Fig 2J). Indeed, spike-gamma LFP correlations were lower 15 minutes after ketamine relative to baseline, saline injection, or the period 75 minutes after ketamine (all *p* < 0.001, paired bootstrap tests; Fig 2J). The concurrent decrease in spike-gamma LFP correlations and increase in gamma power in the MD thalamus suggests that this oscillatory LFP activity reflects an increase in synchronized input to this structure after ketamine administration.

To gain further insight into the relationship between the mPFC and MD thalamus after ketamine, we examined STWAs of spike trains from the MD thalamus referenced to the mPFC LFPs and vice versa. Neither the correlation of mPFC pyramidal spikes referenced to MD thalamus gamma oscillations, nor the correlation of MD thalamus spikes referenced to mPFC gamma oscillations were significantly different from the mean STWA ratio of randomized spike trains before or after ketamine (all *p* > 0.05, permutation tests, n = 82 neurons; S1 Fig). In conjunction with the absence of LFP coherence between mPFC and MD thalamus, these results suggest that mPFC neurons are not directly driving ketamine-induced gamma oscillations in the MD thalamus or vice versa, nor are these oscillations being driven by a common input in the gamma-frequency range.

### Effects of the D4R agonist on gamma power

**Fig 3:**
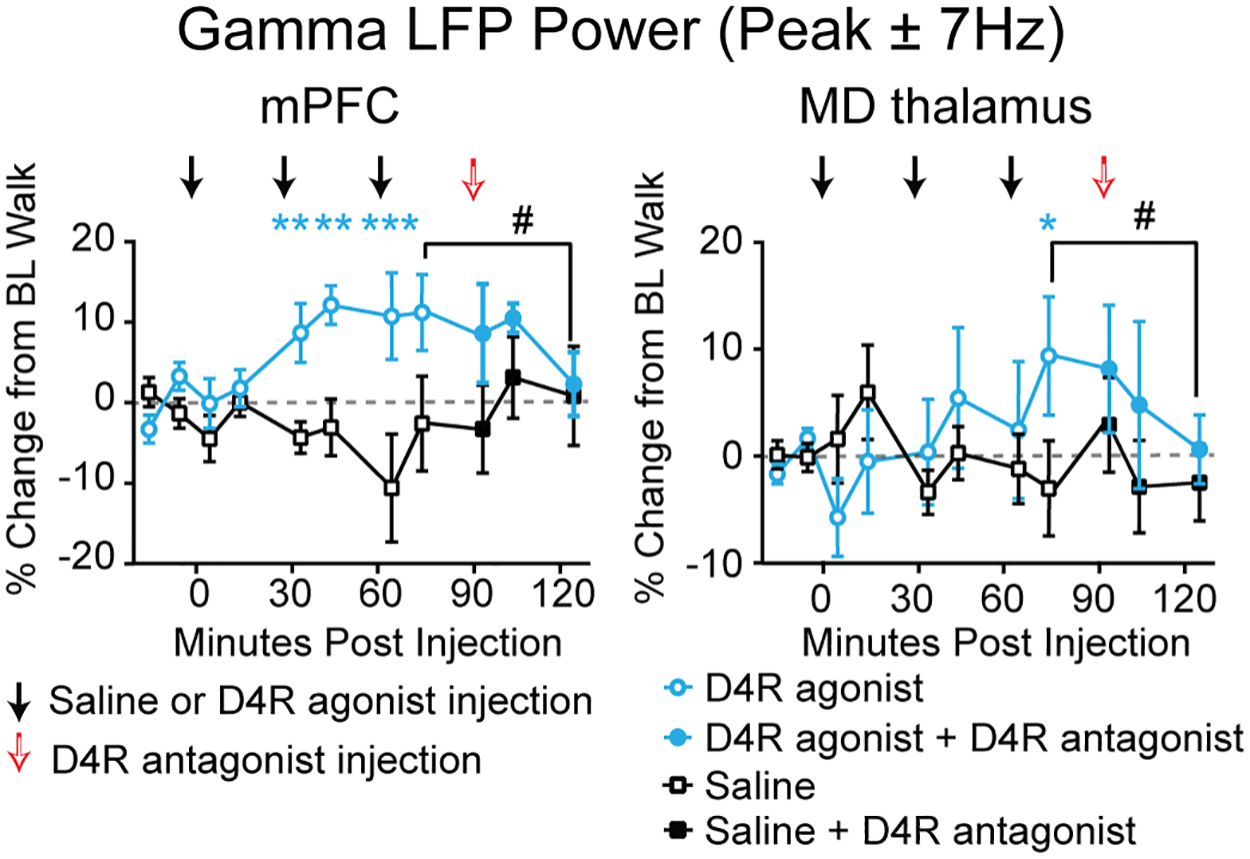
Effects of dopamine D4R agonist and antagonist on gamma power in the absence of ketamine. Percent change in total gamma power (peak between 40 and 70 Hz ± 7 Hz, see Methods) in the mPFC (**left**) and MD thalamus (**right**) of rats injected (at arrows) with either saline (black curve), or the D4R agonist A-412997 (blue curve) at different doses (0.3 mg/kg at 1^st^ arrow; 1.2 mg/kg at 2^nd^ arrow; 1.5 mg/kg at 3^rd^ arrow). The total dose was 3 mg/kg A-412997. The D4R antagonist L-745,870 (5 mg/kg, red arrow, n = 6 rats) was injected 90 min following the first injection of either saline or A-412997. *: *p* < 0.05, ***: *p* < 0.001, significant difference from vehicle injections, repeated measures one-way ANOVA with post-hoc paired bootstrap tests with Bonferroni correction #: *p* < 0.05, paired bootstrap test comparing percent change in power 15 minutes after 3 mg/kg A-412997 and 35 minutes after 5 mg/kg L-745,870.

Dopamine D4R agonists concurrently increase gamma power and cognitive performance in normal animals [45] and D4R antagonists reverse cognitive deficits induced by chronic NMDAR antagonism [47]. To examine the effect of the D4R agonist A-412997 on gamma power during treadmill walking in the absence of ketamine, three ascending doses were administered 30 minutes apart followed by a 5 mg/kg dose of the D4R antagonist L-745,870 (Table 1 and Fig 3). In the mPFC, a total dose of 1.5 mg/kg of A-412997 induced a modest but significant 12.1 ± 2.4% increase in gamma power (*p* < 0.05, paired bootstrap test, n = 6 rats; Fig 3). This was not further increased by a subsequent 1.5 mg/kg injection (total dose of 3 mg/kg A-412997, *p* > 0.05, paired bootstrap test; Fig 3). Likewise, in the MD thalamus, the total dose of 3 mg/kg A-412997 increased gamma power by 9.4 ± 5.5% (*p* < 0.05), but the other doses had no significant effect (*p* > 0.05, repeated measures one-way ANOVA, followed by paired bootstrap tests, n = 6 rats; Fig 3). These increases in gamma power were reduced by the D4R antagonist in the mPFC (-8.9%, *p* < 0.05, paired bootstrap test) and the MD thalamus (-8.8%, *p* < 0.05, paired bootstrap test; Fig 3). However, the D4R antagonist did not significantly change gamma power after vehicle injections (*p* > 0.05, paired bootstrap test; Fig 3). These results are consistent with previous findings showing an increase in prefrontal cortical gamma power with D4R agonists [45,53], and indicate that walking-induced gamma oscillations are not dependent on D4R activity.

### The D4R agonist and antagonist influence ketamine-induced neurophysiological changes in the MD thalamus, but not the mPFC

**Fig 4:**
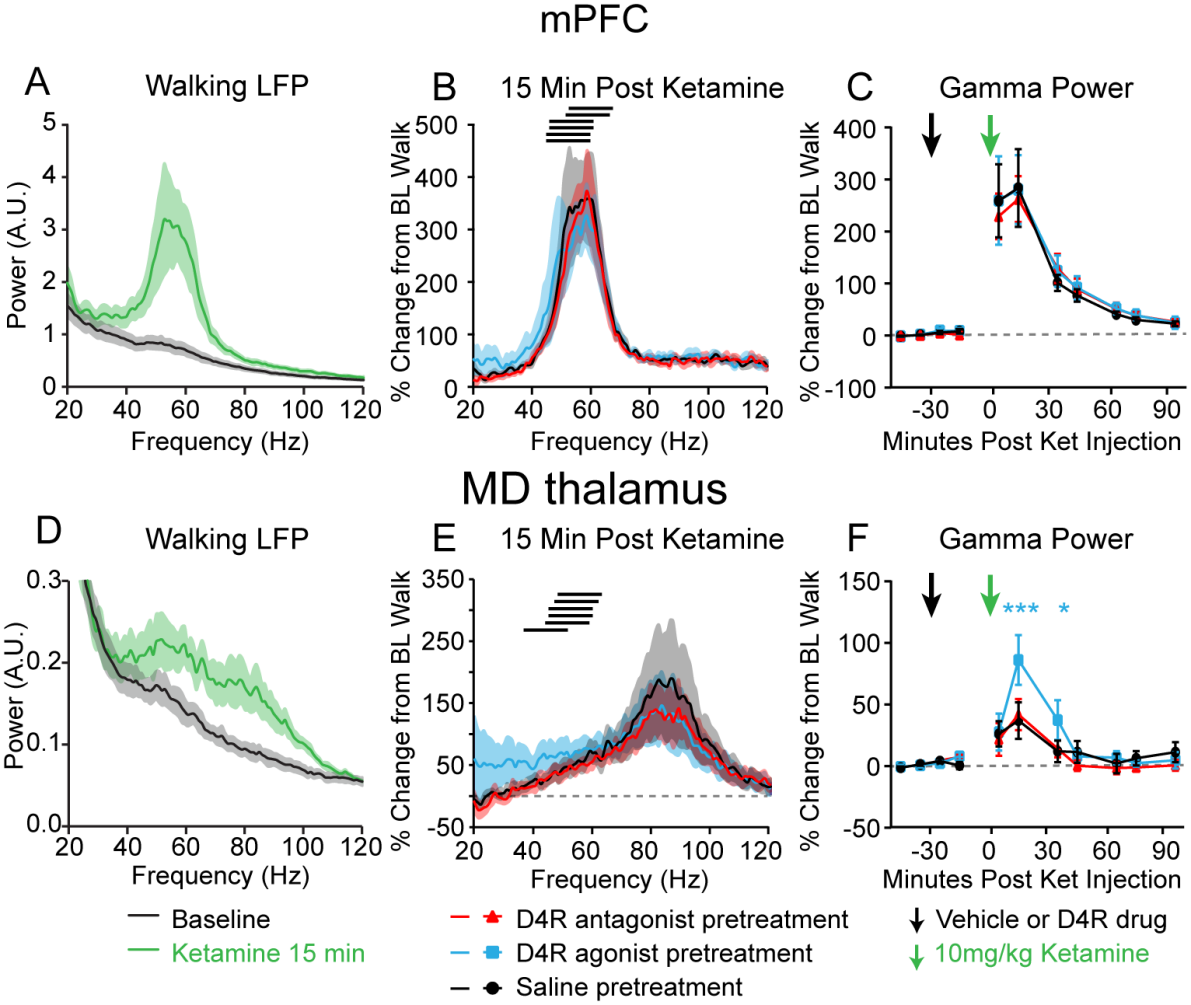
Effects of dopamine D4R agonist and antagonist on ketamine-induced gamma power. **A, D,** Averaged LFP power spectra in the mPFC (**A**) and MD thalamus (**D**) at baseline (black) and 15 minutes after 10 mg/kg ketamine (green) with saline injection 30 minutes prior to ketamine administration (n = 6 rats). **B, E,** Average percent change in the mPFC (**B**) and MD thalamus (**E**) in the LFP power spectra, relative to baseline walking, 15 minutes after rats were injected with 10 mg/kg ketamine. The black bars indicate the gamma frequency range of each individual rat. The red trace indicates pretreatment with the antagonist (5 mg/kg L-745,870), the blue trace indicates the pretreatment with the agonist (3 mg/kg A-412997), and the black trace indicates the pretreatment with saline. All pretreatments were administered 30 minutes prior to ketamine, as shown in (**C**) and (**F**). Black traces are from the ketamine line spectra shown in (**A)** and (**D)** respectively. **C, F,** Changes in low gamma (peak between 40 and 70 Hz ± 7 Hz) power from the frequency ranges shown by the black bars in (**B**) and (**E**) in the mPFC (**C**) and MD thalamus (**F**), taken from 4 minute intervals 10–20 minutes apart, so that data was always taken with the treadmill ON. The pre-treatment injection (black arrow) of saline (black trace), D4R agonist (blue trace) or D4R antagonist (red trace), was administered 30 minutes before the injection of 10 mg/kg ketamine (green arrow). Blue stars indicate a significant change from saline pretreatment with the agonist (n = 6 rats). Data is presented as percent change from the rat’s baseline walking power on the same recording day. Values are reported as percentage ± SEM. **: *p* < 0.01, ***: *p* < 0.001, two-way repeated measures ANOVA followed by paired bootstrap tests with Bonferroni correction.

We next assessed the effects of the D4R agonist (3 mg/kg A-412997 s.c.) and antagonist (5 mg/kg L-745,870 s.c.) administered 30 minutes before a ketamine injection (10 mg/kg s.c.) on ketamine-induced behaviors and gamma power. Neither the D4R agonist nor the D4R antagonist had a significant influence on measures of attentiveness, hyperactivity, stereotypic behaviors, or ataxia after ketamine injection while the treadmill was off or on (all *p* > 0.05, paired bootstrap tests, n = 12 rats, data not shown). Moreover, in the mPFC, the D4R pretreatments neither influenced the low gamma power (peak between 40 and 70 Hz ± 7 Hz) 15 minutes after ketamine administration, nor influenced the time course of gamma power recovery (*p* > 0.05, repeated measures two-way ANOVA, n = 6 rats; Fig 4A–4C). In contrast, in the MD thalamus ketamine-induced increases in gamma power were enhanced by the D4R agonist pretreatment as compared to the saline pretreatment 15 minutes (86.1 ± 20.2% D4R agonist pretreatment vs. 36.9 ± 14.8% saline pretreatment, *p* < 0.001, paired bootstrap test) and 35 minutes after the ketamine injection (37.3 ± 16.1% D4R agonist pretreatment vs. 11.9 ± 8.8% saline pretreatment, *p*<0.05, paired bootstrap test, n = 6 rats; Fig 4D–4F). The D4R antagonist had no significant effect on ketamine-induced low gamma power (*p* > 0.05, repeated measures two-way ANOVA; Fig 4D–4F). These data show that D4R activation influences ketamine-induced gamma oscillation power in the MD thalamus, but not the mPFC.

**Fig 5:**
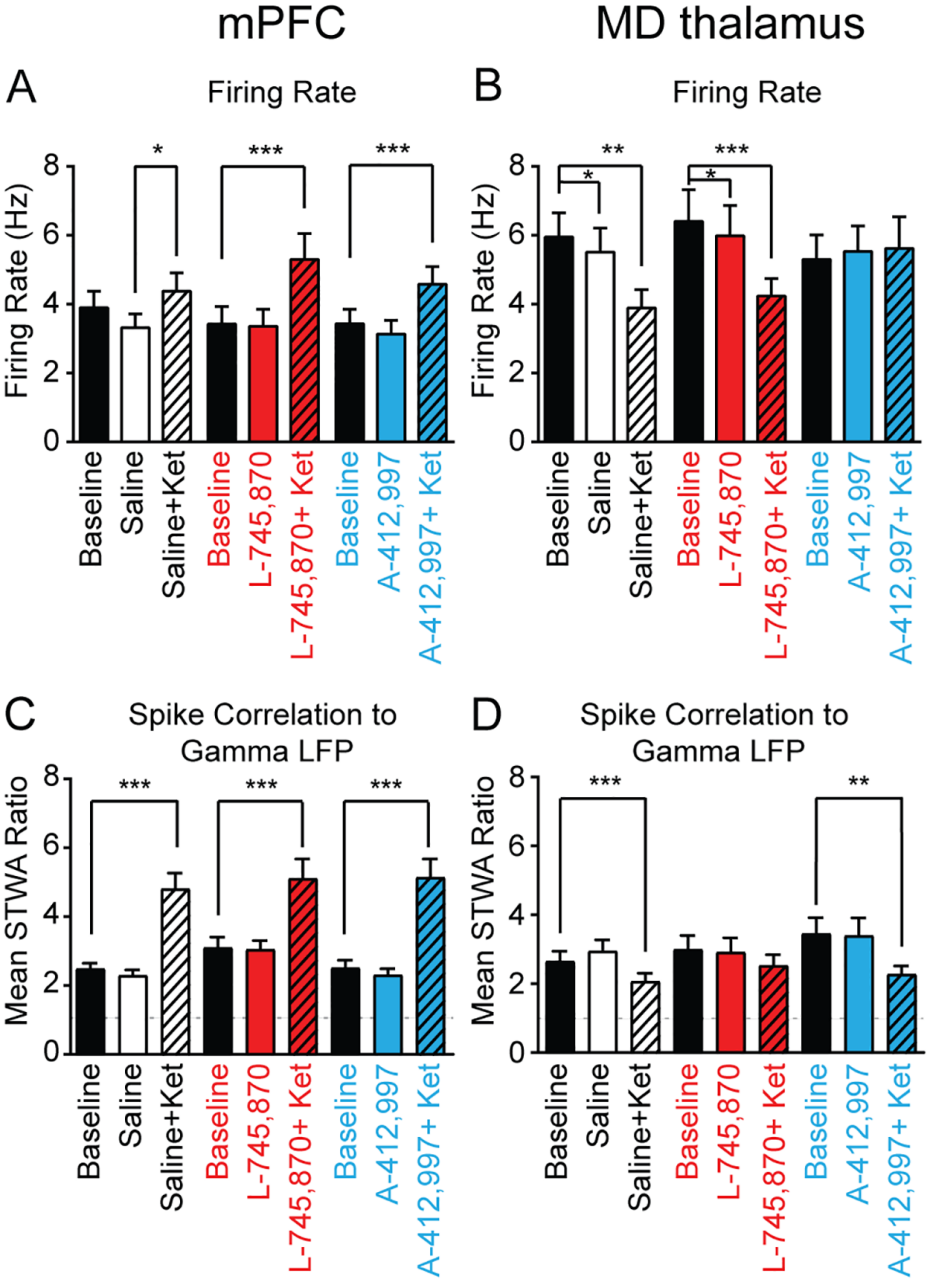
Effects of dopamine D4R agonist and antagonist on ketamine-induced changes in spiking activity. **A-B,** Average firing rates of putative mPFC pyramidal neurons (**A**) and MD thalamus neurons (**B**) at baseline (black), 15 minutes after the pretreatment injection (solid color), and 15 minutes after the subsequent additional administration of 10 mg/kg ketamine (striped). Bars in white, in red or in blue correspond to pretreatments with either saline, antagonist 5 mg/kg L-745,870 s.c., or agonist 3 mg/kg A-412997 s.c., respectively (saline: mPFC: n = 45 neurons, MD thalamus: n = 37 neurons; L-745,870: mPFC: n = 30 neurons, MD thalamus: n = 31 neurons; A-412997: mPFC: n = 38 neurons, MD thalamus: n = 31 neurons). **C-D,** Bar graphs show spike-gamma LFP correlations (filtered from 40–70 Hz) for mPFC pyramidal neurons (**C**) and MD thalamus neurons (**D**). There were no significant differences between pretreatment groups in the effect of ketamine on firing rates or spike-LFP correlations (*p* > 0.05, one-way ANOVAs). All data collected from treadmill walking epochs. Values are reported as mean ± SEM; neurons from 6 rats. *:*p* < 0.05, **:*p* < 0.01, ***:*p* < 0.001, paired bootstrap tests compare baseline walk and the drug treatments.

To assess whether the D4R agonist and antagonist influenced ketamine-induced changes in spiking activity, we examined neurons in the mPFC and MD thalamus after each pretreatment. As described above, ketamine increased firing rates and spike-gamma LFP correlations in the mPFC and decreased both measures in the MD thalamus (Fig 2G–2J). In mPFC pyramidal neurons, no significant differences in ketamine-induced increases in firing rates were observed after pretreatments with either saline, the D4R agonist, or the D4R antagonist (*p* > 0.05, bootstrap tests, saline n = 45 neurons, L-745,870 n = 30 neurons, A-412997 n = 38 neurons; Fig 5A). Furthermore, in the mPFC, the D4R-targeting pretreatments neither influenced ketamine-induced increases in spike-gamma LFP correlation, nor influenced spike-gamma LFP correlation per se (both *p* > 0.05, bootstrap tests; Fig 5C). Interestingly, in the MD thalamus, the D4R agonist pretreatment attenuated the ketamine-induced decreases in neuronal firing rates (saline-ketamine change in firing rates: -2.1 ± 0.6 Hz, n = 37 neurons, vs. D4R agonist-ketamine change in firing rates: +0.3 ± 0.7 Hz, n = 31 neurons, *p* < 0.05, bootstrap test; Fig 5B). Likewise, both the saline pretreatment and the D4R antagonist pretreatment alone induced a modest yet significant decrease in the MD neuronal firing rate of 0.4 ± 0.2 Hz (both *p* < 0.05), but the D4R agonist alone does not (*p* > 0.05, paired bootstrap tests, n = 31 neurons; Fig 5B). Moreover, in the MD thalamus, pretreatment with the D4R antagonist prevented the ketamine-induced desynchronization between spiking and gamma band activity (*p* > 0.05, paired bootstrap test; Fig 5D). Overall, the D4R pretreatments had no effect on ketamine-induced changes in firing rate or spike-gamma LFP correlations in the mPFC, but the D4R agonist modified firing rates before and after ketamine in the MD thalamus. Taken together, these data suggest that the MD thalamus may be more sensitive to perturbations in D4R signaling than the mPFC.

## Discussion

The present results show that acute ketamine administration induces a striking increase in gamma oscillation power in the mPFC and a more modest increase in MD thalamus, while having divergent effects on single neuron activity in these two structures in awake behaving rats. In the mPFC, ketamine increased firing rates and the correlations between mPFC spike timing and gamma oscillations; but in the MD thalamus, ketamine reduced firing rates and spike-LFP correlations in the gamma range. This data is consistent with results from studies in mice showing that ketamine increases glucose metabolism in the PFC and decreases metabolism in the MD thalamus [63,64]. By comparison, treadmill walking alone also increased gamma power in both the mPFC and MD thalamus, but to a lesser extent than ketamine administration and these effects were not additive. In the mPFC, walking concurrently increased firing rates of pyramidal neurons and spike synchronization to gamma oscillations, consistent with previous observations in the visual cortex of mice [65,66]. Moreover, in the MD thalamus, unlike the effect of ketamine, walking concurrently increased the firing rate and synchronization between spikes and gamma oscillations, suggesting that different mechanisms are responsible for walking-induced increases in thalamic gamma power as compared to ketamine-induced increases in thalamic gamma power.

Although dopamine D4R-targeting drugs were ineffective in early clinical trials at treating the positive and negative symptoms of schizophrenia, several lines of evidence have suggested their potential use for ameliorating cognitive deficits in schizophrenia [for a review, see 67]. However, at the concentrations used in the present study, these drugs show limited ability to modulate ketamine-induced gamma activity. In the present study, neither the D4R agonist nor antagonist significantly influenced ketamine-induced gamma oscillations or firing rates in the mPFC. However, in the MD thalamus, the D4R agonist amplified ketamine-induced gamma increases and modified neuronal firing rates, while the D4R antagonist reduced ketamine’s effect on spike-gamma LFP correlations. Currently, little is known about the effects of well-established antipsychotics on the firing activity in the MD thalamus; therefore, it is unclear whether the D4R antagonist’s ability to reduce ketamine’s effects on spike-gamma LFP correlations in the MD thalamus is predictive of potential cognitive therapeutic efficacy.

In addition to ketamine’s use as a model of schizophrenia, there is considerable interest in the use of this drug as an antidepressant [68,69]. Notably, ketamine is used to rapidly treat depression at doses which can induce psychotomimetic symptoms in healthy volunteers (~5–15% of the anesthetic dose); however, the effects of ketamine on depression outlast the duration of the psychotomimetic effects by several days, raising intriguing questions about the mechanism underlying ketamine’s antidepressant activity [70–72]. It remains to be seen whether the current results are relevant to understanding the long lasting therapeutic benefits of ketamine as well as its short-term psychotomimetic effects.

### Neurophysiological correlates of ketamine and locomotion

The present results highlight the observation that ketamine-induced gamma oscillations show similarities to walking-induced gamma oscillations in the mPFC and address the question of whether these two types of gamma are locally generated by a common circuit. In the cortex, volleys of alternating excitation and inhibition from pyramidal neurons and fast-spiking interneurons respectively are assumed to generate gamma LFP power [41,73]. We observed that mPFC pyramidal neurons show increased spike synchronization to gamma oscillations during walking epochs and after ketamine administration concurrent with increases in gamma power, as would be expected in a circuit where power is enhanced by the rhythmic activation of pyramidal neurons [74]. Furthermore, locomotion increased gamma oscillation power in the mPFC with a peak frequency centered at ~51 Hz, consistent with prior work reporting increases in gamma power in the rodent mPFC and visual cortex during locomotion [50,75] and arousal [65,66]. In comparison, ketamine increased gamma power with a peak frequency centered at ~55 Hz, which was similar to, but significantly higher than, the peak observed during locomotion. Although both of these finely-tuned increases in gamma activity may reflect activity in a local circuit with precise temporal dynamics [76], underlying mechanisms remain to be determined.

Other evidence suggests a more complex mechanism of gamma power generation after ketamine administration in the mPFC. Acute application of NMDA receptor antagonists increases gamma power, and increases the firing rates of pyramidal neurons, but decreases the firing rates of interneurons over a similar time course [35,36, for a review, see 77]. For the disinhibition of pyramidal neurons to directly increase gamma power, one would expect these neurons to exhibit increased synchronization with gamma oscillations [78,79]. In agreement with this, our results showed that ketamine administration increased the firing rates and spike-gamma LFP correlations of mPFC pyramidal neurons. Interestingly, the increases in firing rate and gamma power were not proportional. While pyramidal neurons showed similar increases in firing rate induced by ketamine or walking (33% vs. 11%), increases in gamma power induced by ketamine were substantially larger than those induced by walking in the mPFC (254% vs. 23.8%). However, subtle changes in spike timing appear to be critical in inducing changes in LFP power as the spike-gamma LFP correlations better reflected the magnitude of changes in gamma power after both ketamine and walking (107% vs. 22% respectively) than did firing rates. Similarly, Wood and colleagues [37] observed a lower correlation between firing rates and gamma power in the frontal cortex after administration of the NMDAR antagonist MK-801. Furthermore, while we observed increased spike-gamma LFP synchronization after ketamine treatment, Molina and colleagues reported that MK-801 treatment in rats disorganized action potential firing while increasing gamma power in the prefrontal cortex [40]. Thus, the relationship between pyramidal neuron firing rate and abnormal gamma power after ketamine administration remains complex, consistent with its effects on the excitatory/inhibitory balance in the cortex [3].

While considerable interest has focused on the role of the PFC in the etiology of schizophrenia [80], alterations in thalamic activity have also been implicated [81–83], with a special emphasis on the MD thalamus [11,84,85]. Additionally, rodent studies have shown that reductions in neuronal activity in the MD thalamus interfere with cortical synchrony [34] and working memory [86,87], and human studies suggest that reduction in thalamic activity may be related to the emergence of cognitive or psychotomimetic symptoms [14,88]. In the present study, we observed that ketamine increased gamma power in the MD thalamus, comparable to increases observed in the rodent ventrolateral thalamus or ventroposteriomedial thalamus [31,32], and human thalamus [20]. However, increases in gamma power emerged over a broader range in the MD thalamus than in the mPFC, with two spectral peaks centered at 55 Hz and 81 Hz. Although mPFC and MD thalamus are reciprocally connected [89,90] and both show gamma peaks around 55 Hz, ketamine did not induce a significant increase in gamma LFP coherence, or increase spike-gamma LFP synchronization between these structures. Consistent with our observations, ketamine does not appear to alter the functional connectivity between the MD thalamus and PFC in humans [18]; on the other hand, studies using other measures of connectivity involving other thalamic nuclei have shown that ketamine increases thalamocortical connectivity [20,32,91]. In addition to the increases in gamma power in the MD thalamus, ketamine simultaneously decreased firing rates and reduced correlations between spikes and gamma LFP in the MD thalamus, suggesting that the observed increase in gamma power is driven by external inputs [92,93]. Our observation that ketamine decreased the firing rates of MD thalamus neurons *in vivo* is consistent with *in vitro* results testing NMDA antagonists in ventrobasal thalamus neurons [94]. Given that the majority of excitatory neurotransmission in the MD thalamus is mediated through NMDA receptor signaling [95], it is unsurprising that antagonizing NMDA receptors via ketamine administration reduces the average firing rate of MD thalamus neurons. Similarly, ketamine reduces glucose metabolism in the MD thalamus of mice [63,64], consistent with a reduction in neuronal activity. Nevertheless, another NMDAR antagonist, phencyclidine, has been reported to reduce the firing rate of inhibitory thalamic reticular neurons [96] and increase the firing rate of ~57% of MD thalamus neurons [39]. Overall, however, our results support the idea that reduced spiking activity in the MD thalamus may be associated with psychotomimetic symptoms induced by ketamine.

### Behavioral changes after ketamine

While subanesthetic doses of ketamine have served as a translational model for early stages of schizophrenia due to its psychotomimetic and cognitive effects [27], they have not been used to model changes in motor activity associated with schizophrenia. This is because motor alterations associated with schizophrenia range from catatonia (associated with negative symptoms) to agitation (associated with positive symptoms) and abnormal involuntary movements [97]. Furthermore, while subanesthetic doses of ketamine induce an increase in motor activity in rodents, they do not appear to induce hyperactivity or agitation in healthy humans [98]. Nevertheless, the treadmill design allowed us to examine how ketamine affected treadmill walking. We observed that rats collided with the paddle within the treadmill less frequently after 10 mg/kg ketamine than at baseline, suggesting that ketamine induced an increase in attention during the walking task. This supports the idea that gamma power and attention are correlated [99], even when induced by ketamine, and is consistent with a recent study showing that ketamine increases attention in young rodents [100].

### Dopamine D4 receptor

The ability of a drug to modulate ketamine-induced gamma power has been proposed as a useful index for assessing the therapeutic potential of drugs in treating cognitive deficits in patients with schizophrenia [27,5#4]. Low doses of D4R antagonists reverse working memory deficits induced by stress or chronic PCP administration in monkeys [46,47], but decrease task performance when acutely co-administered with the antipsychotic clozapine or lurasidone [44]. Also, D4R agonists have the ability to increase gamma power and cognitive performance in normal animals [43,45,53,101–104]. Furthermore, humans with an allelic variant of the D4R (D4.7) with lower dopamine responsiveness show abnormal gamma oscillatory activity during a task requiring attention [105], and have a higher incidence of attention deficit hyperactivity disorder (ADHD) [106]. Therefore, both the D4R agonist and antagonist have potential pro-cognitive effects that we sought to further explore in the modulation of ketamine-induced gamma power.

As ketamine administered acutely increases extracellular dopamine in the cortex of rodents [107], and the D4R has the highest affinity for dopamine of any dopaminergic receptor [108,109], we hypothesized that ketamine administration would activate D4Rs; therefore, the D4R antagonist would diminish ketamine’s effects. However, pretreatment with the D4R antagonist had little effect. Instead, pretreatment with the D4R agonist augmented ketamine’s effects on gamma power and reduced ketamine’s effects on firing rates in the MD thalamus. Other investigators have shown changes in cortical ketamine-induced gamma power using chronic, but not acute, pretreatment with antipsychotics [54,110,111]. Thus, it remains to be seen whether chronic treatment with D4R-targeting drugs could reduce ketamine-induced gamma power. The selective effects of a D4R agonist on ketamine-induced gamma power in the MD thalamus could be attributable to region-specific differences in D4R expression. Indeed, in the cortex [42] and the hippocampus [43] D4R expression is highest in parvalbumin-positive fast-spiking interneurons, however the D4R is also sparsely expressed in pyramidal neurons [42]. Although it is unclear the extent to which the thalamic reticular nucleus projects to the MD thalamus in rats [112], given that presynaptic inputs into the thalamic reticular nucleus express D4Rs [42,113,114], the thalamic reticular nucleus could provide D4R-mediated modulation to the MD thalamus.

### Conclusion

Overall, the present study demonstrates that different mechanisms appear to underlie ketamine-induced increases in gamma oscillation power in the mPFC and MD thalamus during treadmill walking, but leave unresolved the nature of the relationship between the two areas. Nevertheless, the observation that ketamine affects LFPs and spike-gamma LFP relationships in the MD thalamus as well as in the mPFC suggests that the MD thalamus may also contribute to ketamine-induced psychotomimetic and cognitive symptoms related to early stage schizophrenia.

## Supporting information

S1 Table Experimental design. Randomized drug delivery schedules for each group of rats, with 1 week washout between recordings. Rats in the first two groups were randomly assigned to receive the D4R agonist or the vehicle dose response curve on week 1, and rats in the second and third groups received the ketamine pretreatments, 1 mL/kg saline, the D4R agonist 3 mg/kg A-412997, and the D4R antagonist 5 mg/kg L-745870 in a randomized order.
(DOCX)

S2 Table Behavioral measures before and after ketamine during treadmill on and off epochs. Quantified measures of behavior from ~15 minutes before and after ketamine administration. Values are reported as percentage or mean ± SEM. *: *p* < 0.05, **: *p* < 0.01, ***: *p* < 0.001, paired bootstrap tests.
(DOCX)

S1 Fig Coherence between the mPFC and MD thalamus. **(A)** Line graphs show averaged coherence spectra (0.7–120 Hz) of drug naïve rats with the treadmill on before ketamine administration (black, n = 5 rats) and 15 minutes after ketamine administration (green). Colored shadows indicate SEM. Raw LFP traces were smoothed and downsampled to 250 Hz before coherence was calculated. **(B)** Bar graphs show the mean STWA ratio of mPFC neurons referenced to the LFP of the MD thalamus (left) and MD neurons referenced to the LFP of the mPFC (right). STWA ratios are the ratio of the peak-to-trough amplitude of the unshuffled STWA/shuffled STWA of spike trains to LFPs. A STWA ratio close to 1 indicates that a spike train’s relationship to ongoing oscillations resembles a randomly shuffled spike train.
(TIF)

S2 Fig mPFC and MD thalamus gamma range phase difference histograms. **(A)** Polar plots depict phase-difference histograms between mPFC and MD thalamus LFPs filtered to the 40–70 Hz frequency range in drug naïve rats during rest and treadmill walking. The radial axis depicts the number of observations of phase differences in each bin. **(B)** Polar plots depict gamma-range phase-difference histograms before and after ketamine administration during treadmill walking. Note that each phase-difference histogram does not show a significant phase difference preference, nor do any exhibit a multi-modal distribution. These results concur with the measurements of FFT-spectral coherence in each state, namely that there does not appear to be any significant phase correlation between the mPFC and MD thalamus in the gamma frequency range during these behaviors.
(TIF)

## Abbreviations

D4R: Dopamine D4 receptor
i.p.: intraperitoneal
LFP: local field potential
MD: mediodorsal thalamus
mPFC: medial prefrontal cortex
NMDA: N-methyl-D-aspartate
s.c.: subcutaneous
STWA: spike-triggered waveform average
