# Supplementary material for: Neuronal correlates of ketamine and walking induced gamma oscillations in the medial prefrontal cortex and mediodorsal thalamus
Source: PLoS One. 2017 Nov 2;12(11):e0186732. doi: 10.1371/journal.pone.0186732 (PMC5667758; doi:10.1371/journal.pone.0186732)
Supplement: S1 Table — Randomized drug delivery schedules for each group of rats, with 1 week washout between recordings. Rats in the first two groups were randomly assigned to receive the D4R agonist or the vehicle dose response curve on week 1, and rats in the second and third groups received the ketamine pretreatments, 1 mL/kg saline, the D4R agonist 3 mg/kg A-412997, and the D4R antagonist 5 mg/kg L-745870 in a randomized order. (DOCX) [file pone.0186732.s001.docx]

**S1 Table. Experimental design.**

| Group | Week 1 | Week 2 | Week 3 | Week 4 | Week 5 | Week 6 |
| --- | --- | --- | --- | --- | --- | --- |
| N = 4 rats | D4R/vehicle dose response curves: rats randomly assigned to receive vehicle of D4R agonist first | | D4R antagonist dose response (data not shown) | Ascending doses of ketamine (data not shown) | Sacrificed |  |
| N = 2 rats | D4R/vehicle dose response curves: rats randomly assigned to receive vehicle of D4R agonist first | | Vehicle, A412997, or L745870 pretreatment 30 minutes before ketamine injection: order of treatments randomly assigned | | | Sacrificed |
| N = 4 rats | Double doses of ketamine (data not shown) | Vehicle, A412997, or L745870 pretreatment 30 minutes before ketamine injection: order of treatments randomly assigned | | | Sacrificed |  |

**S1 Table. Experimental design.** Randomized drug delivery schedules for each group of rats, with 1 week washout between recordings. Rats in the first two groups were randomly assigned to receive the D4R agonist or the vehicle dose response curve on week 1, and rats in the second and third groups received the ketamine pretreatments, 1 mL/kg saline, the D4R agonist 3 mg/kg A-412997, and the D4R antagonist 5 mg/kg L-745870 in a randomized order.
