# Supplementary material for: Neuronal correlates of ketamine and walking induced gamma oscillations in the medial prefrontal cortex and mediodorsal thalamus
Source: PLoS One. 2017 Nov 2;12(11):e0186732. doi: 10.1371/journal.pone.0186732 (PMC5667758; doi:10.1371/journal.pone.0186732)
Supplement: S2 Table — Quantified measures of behavior from ~15 minutes before and after ketamine administration. Values are reported as percentage or mean ± SEM. *: p < 0.05, **: p < 0.01, ***: p < 0.001, paired bootstrap tests. (DOCX) [file pone.0186732.s002.docx]

**S2 Table. Behavioral measures before and after ketamine during treadmill on and off epochs.**

|  | Behaviors | Pre-ketamine | Post-ketamine |
| --- | --- | --- | --- |
| Treadmill-OFF Behaviors | | | |
| Hyperactivity | Time moving trunk or limbs (seconds) | 1.3 ± 0.7 | 45.8 ± 0.9*** |
|  | Turns | 0.1 ± 0.1 | 2.4 ± 0.6** |
|  | Time walking backwards (seconds) | 0 ± 0 | 2.0 ± 0.7* |
| Stereotypy | Head-bobs | 0.3 ± 0.2 | 3.3 ± 0.8** |
| Ataxia | Ataxia Score (0-4) | 0 ± 0 | 1.3 ± 0.2*** |
| Treadmill-ON Behaviors | | | |
| Hyperactivity | Turns | 0 ± 0 | 0.6 ± 0.3 |
| Attentiveness | Paddle Hits | 8.7 ± 2.0 | 3.0 ± 0.6* |
|  | Time touching paddle (seconds) | 9.9 ± 2.4 | 4.8 ± 1.7 |
|  | Time per paddle hit (seconds) | 0.9 ± 0.2 | 2.0±0.9 |

**S2 Table. Behavioral measures before and after ketamine during treadmill on and off epochs.** Quantified measures of behavior from ~15 minutes before and after ketamine administration. Values are reported as percentage or mean ± SEM. *: *p* < 0.05, **: *p* < 0.01, ***: *p* < 0.001, paired bootstrap tests.
