# Supplementary figures and images for: Neuronal correlates of ketamine and walking induced gamma oscillations in the medial prefrontal cortex and mediodorsal thalamus
Source: PLoS One. 2017 Nov 2;12(11):e0186732. doi: 10.1371/journal.pone.0186732 (PMC5667758; doi:10.1371/journal.pone.0186732)

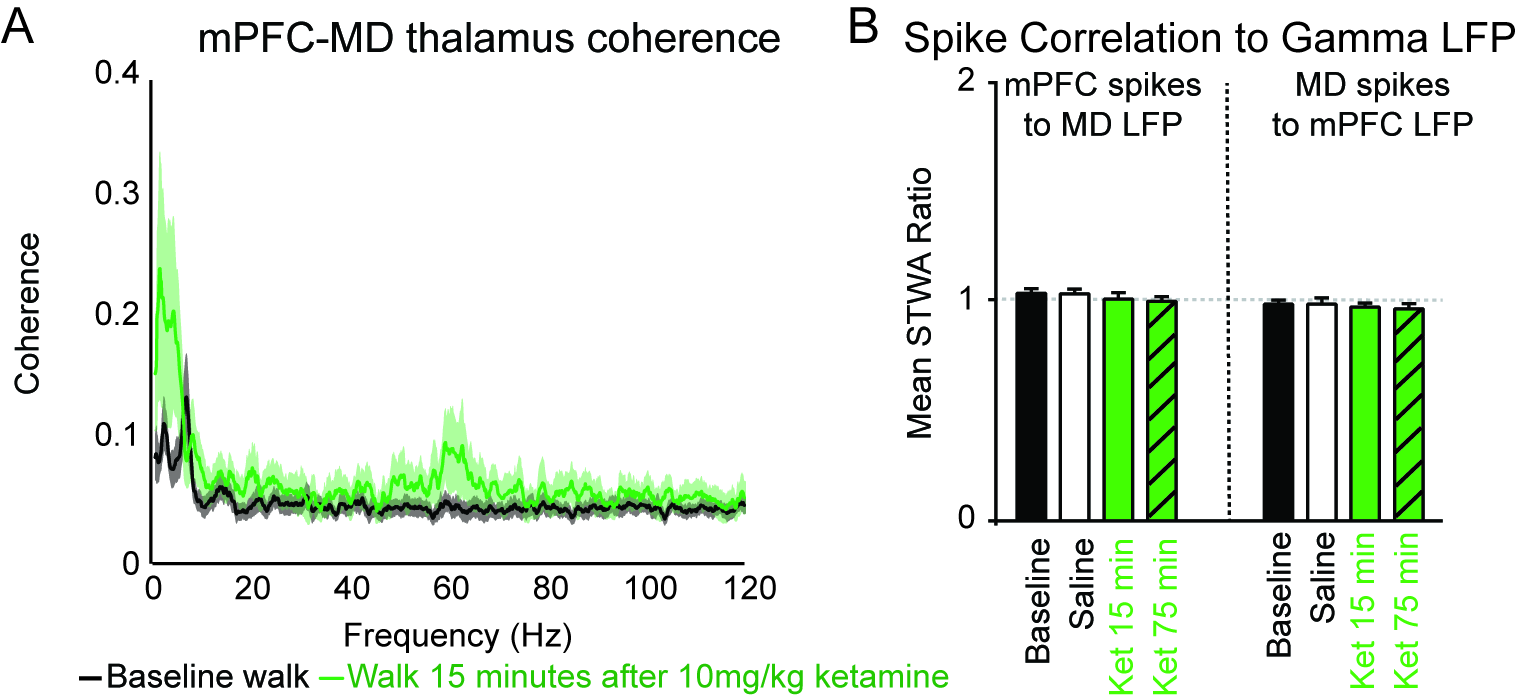

Supplement: S1 Fig — (A) Line graphs show averaged coherence spectra (0.7–120 Hz) of drug naïve rats with the treadmill on before ketamine administration (black, n = 5 rats) and 15 minutes after ketamine administration (green). Colored shadows indicate SEM. Raw LFP traces were smoothed and downsampled to 250 Hz before coherence was calculated. (B) Bar graphs show the mean STWA ratio of mPFC neurons referenced to the LFP of the MD thalamus (left) and MD neurons referenced to the LFP of the mPFC (right). STWA ratios are the ratio of the peak-to-trough amplitude of the unshuffled STWA/shuffled STWA of spike trains to LFPs. A STWA ratio close to 1 indicates that a spike train’s relationship to ongoing oscillations resembles a randomly shuffled spike train. (TIF) [file pone.0186732.s003.tif]

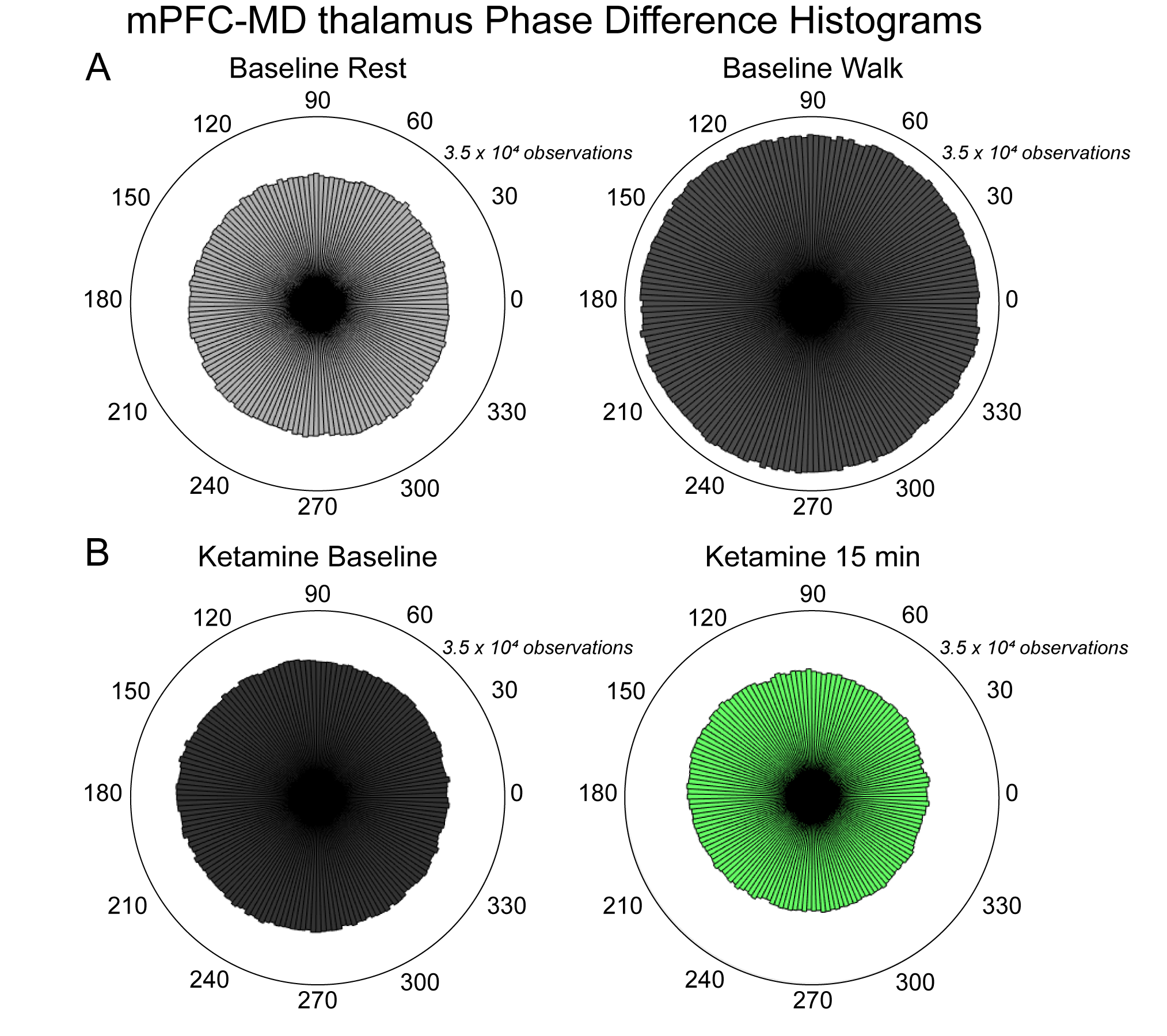

Supplement: S2 Fig — (A) Polar plots depict phase-difference histograms between mPFC and MD thalamus LFPs filtered to the 40–70 Hz frequency range in drug naïve rats during rest and treadmill walking. The radial axis depicts the number of observations of phase differences in each bin. (B) Polar plots depict gamma-range phase-difference histograms before and after ketamine administration during treadmill walking. Note that each phase-difference histogram does not show a significant phase difference preference, nor do any exhibit a multi-modal distribution. These results concur with the measurements of FFT-spectral coherence in each state, namely that there does not appear to be any significant phase correlation between the mPFC and MD thalamus in the gamma frequency range during these behaviors. (TIF) [file pone.0186732.s004.tif]
